# Supplementary material for: The spatio-temporal architecture of everyday manual behavior
Source: Sci Rep. 2023 Jun 9;13:9451. doi: 10.1038/s41598-023-36280-4 (PMC10256758; doi:10.1038/s41598-023-36280-4)
Supplement: Supplementary file 1 — Supplementary Information. [file 41598_2023_36280_MOESM1_ESM.pdf]

The spatio-temporal architecture of everyday manual behavior

Authors: Daniele Sili[1,2], Chiara De Giorgi[1,2]§, Alessandra Pizzuti[1,2]§, Matteo Spezialetti[1,2†], Francesco de Pasquale[3], Viviana Betti[1,2*]

affiliations: [1]Department of Psychology, Sapienza University of Rome, Italy; [2]IRCCS Fondazione Santa Lucia, Rome, Italy, [3]Faculty of Veterinary Medicine, University of Teramo, Teramo, Italy.

* Corresponding author: viviana.betti@uniroma1.it
§ now, at a different institution
† now, at Department of Information Engineering, Computer Science and Mathematics, University of L'Aquila

Supplementary Information

Descriptive statistics

In this section, we present a set of basic statistics of hand kinematics associated with naturalistic grasping movements recorded with the Cyberglove II. In line with previous works (*e.g.,* [1]), we provided the maximum, minimum, mean, median, range, standard deviation and skewness of all sensors averaged across the subjects (see Table S2). Across subjects, the average duration of the experiment was 508 seconds.

Methodological considerations

In this work, we propose a complex stepwise analysis pipeline to extract hand states. This approach is based on the choice of a short time window (2s). Such a choice was driven by the adopted experimental paradigm. In fact, one of the novelties of this work is that we studied unconstrained and not-aligned tasks. To this aim, we need to exploit a sufficiently high temporal resolution to follow the different phases of the movements. This is different from the existing literature, where participants typically perform stereotyped and controlled movements, and thus PCA can be run on a

large number of samples and trials. Thus, the choice of the window length plays a fundamental role and two important aspects regarding the window length must be considered. First, if the window length is too small as compared to the time scale of the movements, we might capture just small fluctuations around static postures that can be considered as noise. Second, even if the window size is appropriate, methodologically PCA might not have enough samples to produce reliable results. As far as it regards the scale of the considered movements, we based our choice on previous studies. For example, Castiello and colleagues (2005) in studying reach-to-grasp movements showed that different phases of the grasping, *i.e.,* from the opening of the hand to its gradual closure around the object, happen within 1.2-1.5-second windows [2]. Since we are interested, not only in the hand pre-shaping, but also in the phases of hand-object interaction, we adopted 2-second windows. However, in our experiment we might have temporal instances corresponding to static postures. The point is how often these happen during the experiment and thus their impact on the PC estimation. To address this point, for each subject and sensor, we extracted the range of the movement, within all the extracted 2s windows. The idea is that, if these windows capture only noise fluctuations around a static position, the range distribution should be highly peaked around zero, *i.e.*, showing the predominance of extremely small values. In Fig. S1a, we report, for a representative subject, the variation over time (left panel) and the distribution (right panel) of the range values for the most active (Middle metacarpophalangeal joint - top) and the least active sensor (Little-Ring abduction joint - bottom). Of note, from the temporal dynamics of the range (graphs on the left) we do not observe large temporal lags where negligible movements (extreme small values of range) are consistently observed. This suggests that the hand does not dwell in static positions for long periods; small movements are continuously interleaved with large ones. In terms of distribution of these values, as it can be noted in Fig. S1a (right panels), for the most active sensor the mean value is 48 deg. For the least active sensor, the mean value is 5.8 deg. Now, even in this case, we note that the distribution is not peaked around extremely small values but a fair proportion of them falls close to the mean (black vertical line). Since PCs represent the weighted contribution of each joint within

a synergistic movement, in Fig. S1b, we show the distribution of the range averaged across all sensors. In this case, the mean value is 27 degrees, which corresponds to meaningful movements. The distribution seems fairly symmetric around the mean, showing the absence of a predominant proportion of minimal movements. Finally, to test whether this window size provides a sufficient number of samples for PCA, we tested our analysis on a controlled experiment. In this case, participants performed stereotypical sequential hand movements (opening-closure and pinch) repeated eight times for 2 seconds. Our results show that the obtained synergies accurately reproduced the controlled hand configurations (see Fig. 3). This suggests that the window size is methodologically appropriate.

Although a systematic analysis of the impact of the window size is beyond the scope of this study, as a control, we tested the adoption of a window size of 4 seconds. As it can be seen in Fig. S6 the dynamics of hand states obtained with 2s and 4s windows lengths are similar.
Consistently with previous results, we obtained 7 states; among them, five states coincided with the previous ones while two states (S3 and S6) are slightly different. However, at the temporal level we observed some interesting differences. As expected, the temporal dynamics (Fig. S6 panel a,c) of these states, represents a slower version of the previous one. However, this slower dynamic seems to miss some important transitions. In fact, as shown in Fig. S6d, in the transition matrix, the largest values are observed along the diagonal. Thus, the adoption of a 4s window seems to lead to approximately the same states with slower dynamics where transitions among states are often missed.

Another methodological consideration regards the developed analysis pipeline which is composed of several consecutive steps. This is the result of preliminary analyses where several techniques and approaches have been tested. Due to the complexity of this dataset, acquired during naturalistic movements, automatic techniques, able to extract synergies directly from the data, failed. For this

reason, we broke down our analysis into distinct steps to address specific experimental questions separately and at incremental level of complexity:

- to identify PCs explaining a large amount of variance
- to group together PC based on their pattern within each subject
- to identify consistent PC recurrent across subjects.

The final hand states recapitulate synergies that explain a large amount of variance, correspond to the same spatio-temporal patterns and are recurrent across subjects.

In the development of the analysis, we tested Hidden Markov Models (HMM) that are excellent candidates to extract spatio-temporally hand states. We adopted HMM to segment the recorded data into a discrete set of functional states that recur over time and highlight invariant aspects of motion. However, the first needed parameter for HMM is the number of states. Unfortunately, in our case, various statistical predictors such as BIC and AIC, failed to converge to a finite number of states. Another important methodological aspect is the definition of "hand states "states". To map synergies into a hand state might be confusing. Synergies have a dynamic meaning representing a spectrum of postures. Specifically, each synergy is represented by a set of weights and their temporal evolution. Thus, theoretically, synergies can be clustered by using both the spatial and temporal information. However, since in our experiment movements are self-paced and not temporally aligned, we could not directly exploit the temporal patterns of the synergies in the clustering both within and across subjects. Here we assumed that each synergy could be represented by the centroid of the underlying movement and we tried to identify their similarities across subjects. As if in a movie we used a representative frame of the underlying action.

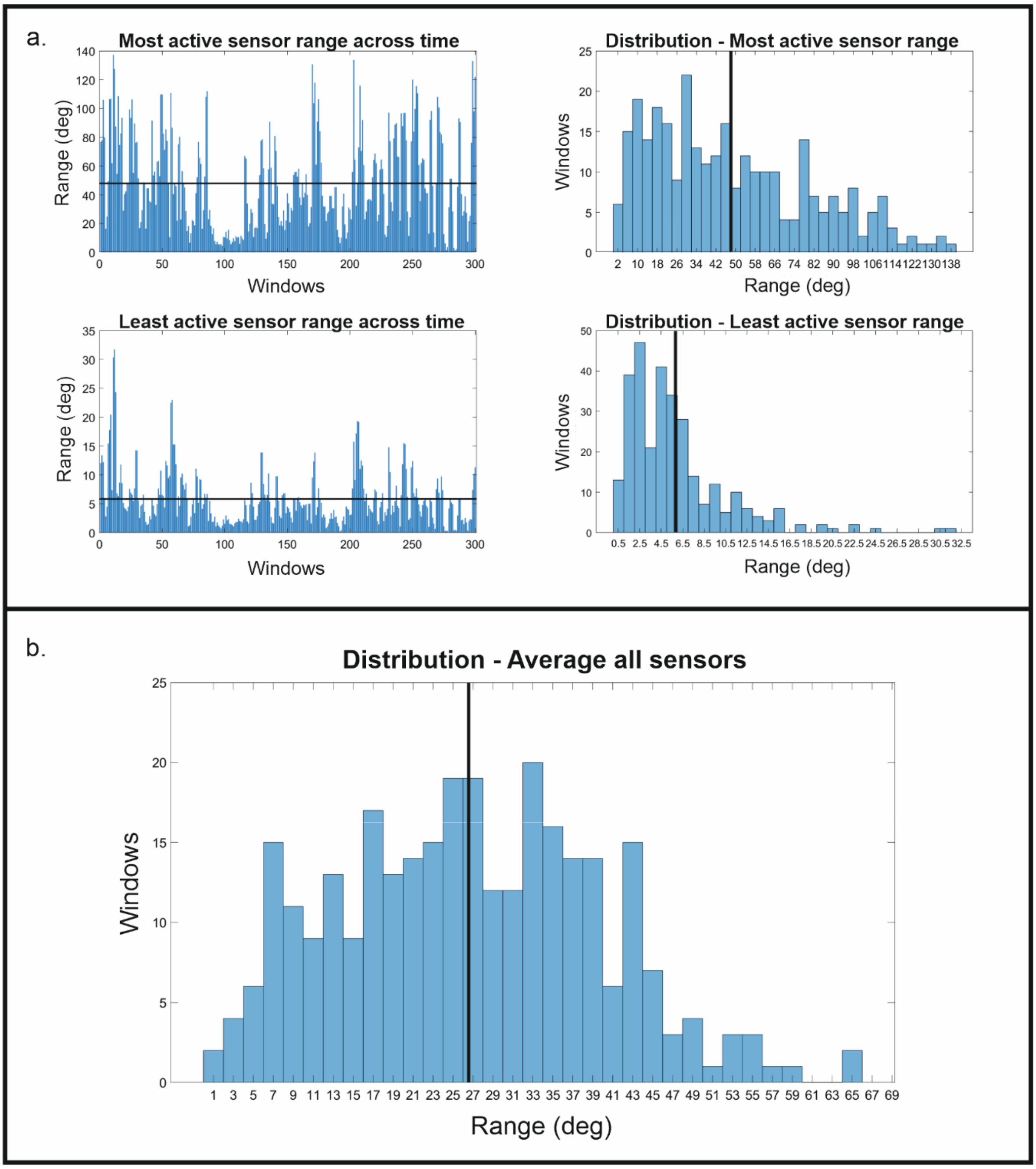

Figure S1. Range of the movement and distribution within the 2s windows. (a) Range values for the most active (Middle metacarpophalangeal joint - top) and the least active sensor (Little-Ring abduction joint - bottom). The variation over time (on the left) and the distribution (on the right) of the range are shown for a representative subject. The temporal dynamics show that the hand does not dwell in static positions for long periods but small movements are interleaved with larger ones continuously. The black horizontal (left panels) and vertical (right panels) line represents the average value: around 48 degrees for the most active sensor and around 5.8 for the least. (b)

Distribution of the range averaged across all sensors. It can be noted a mean value across the sensors of 27 degrees.

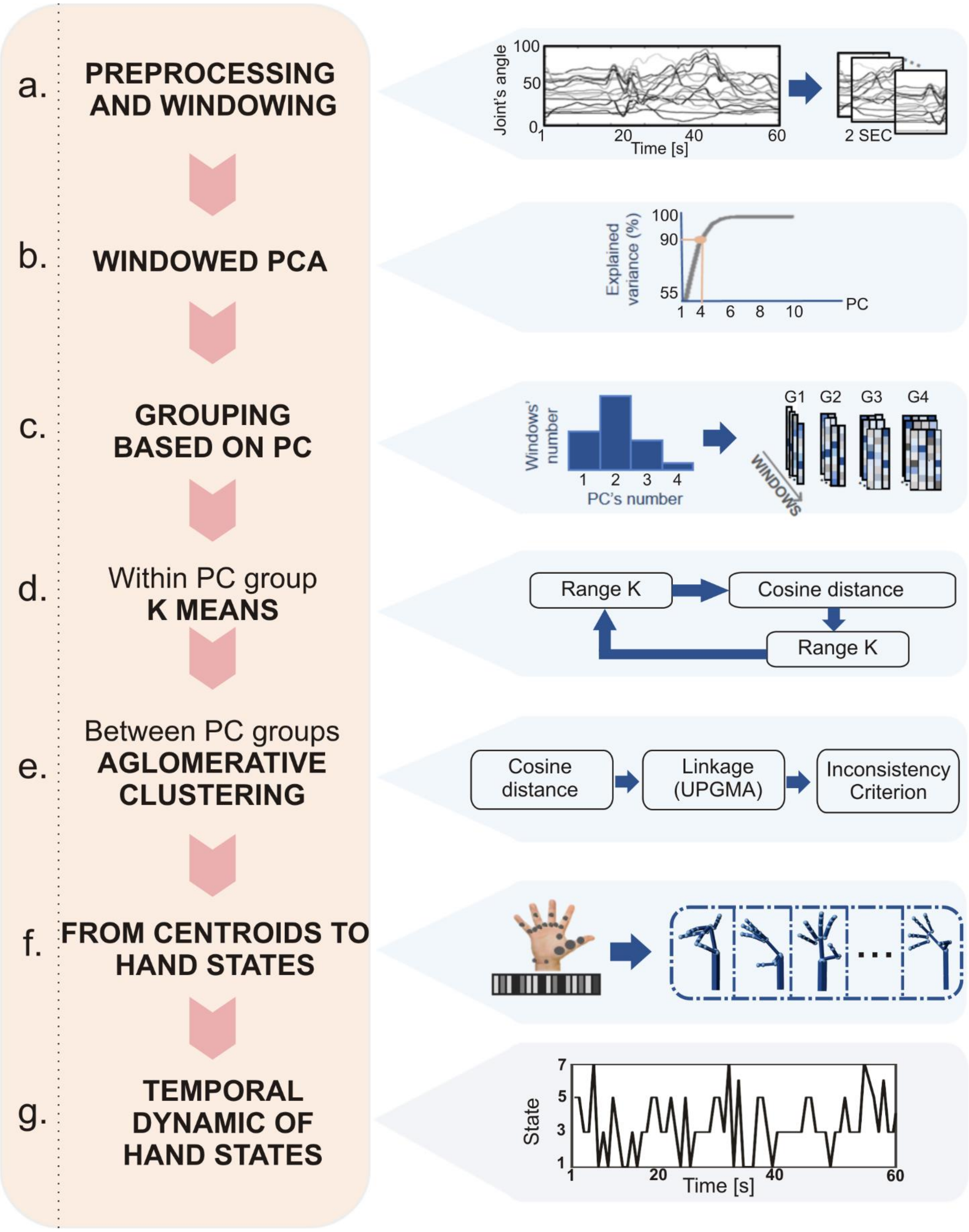

Figure S2. Analysis pipeline. (a) Preprocessing and windowing: the acquired kinematic data are interpolated and calibrated. A low pass FIR filter is applied, and the signals are sampled with overlapping windows. (b) Hand kinematics has a low dimensionality: for each participant, in every window, PCA is applied to extract components accounting for at least 90% of the observed variance. A number of 4 PCs explained at least 90% of the variance. (c) Group analysis across all subjects: the histogram shows for all subjects 4 PCs are enough to explain at least 90% of the variance. On the right panel, we report for each window the eigenvectors that form the 4 PC groups for a representative participant. (d) Within group clustering: K-means clustering algorithm within PC groups across the time-windows. The optimal number of classes is provided by VRC (see Materials and Methods). (e) Between group clustering: hierarchical cluster analysis on all the centroids obtained from the K-means. (f) Final K-means clustering for the hand states: centroids of all participants were clustered to obtain a common set of hand shapes. Each 17-dimensional final centroid was mapped with a Simulink hand model (g) Temporal dynamics of hand states.

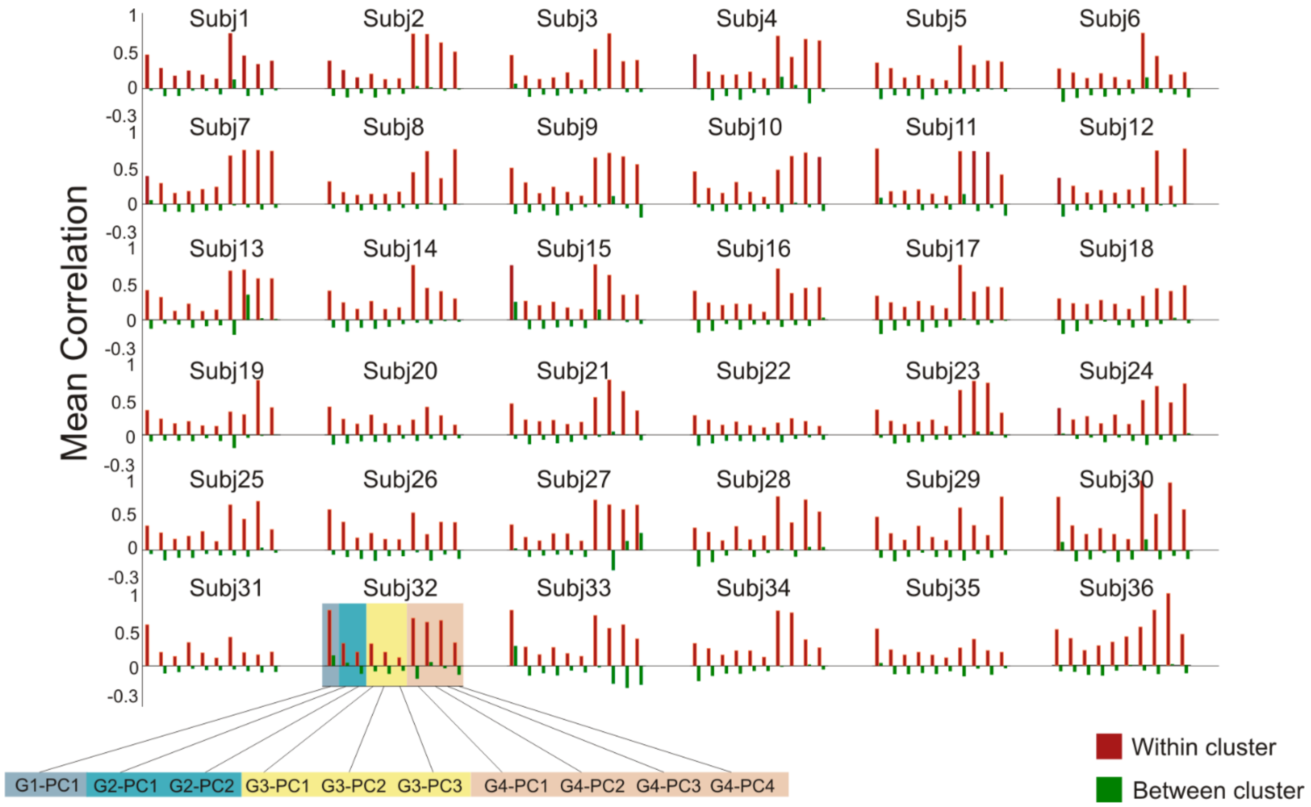

Figure S3. Mean correlation within and between clusters for each group and participant. The mean correlation within clusters (red bars) is significantly stronger than the correlation between the clusters (green bars). This effect is tested on all subjects using a t-test (α=0.05)

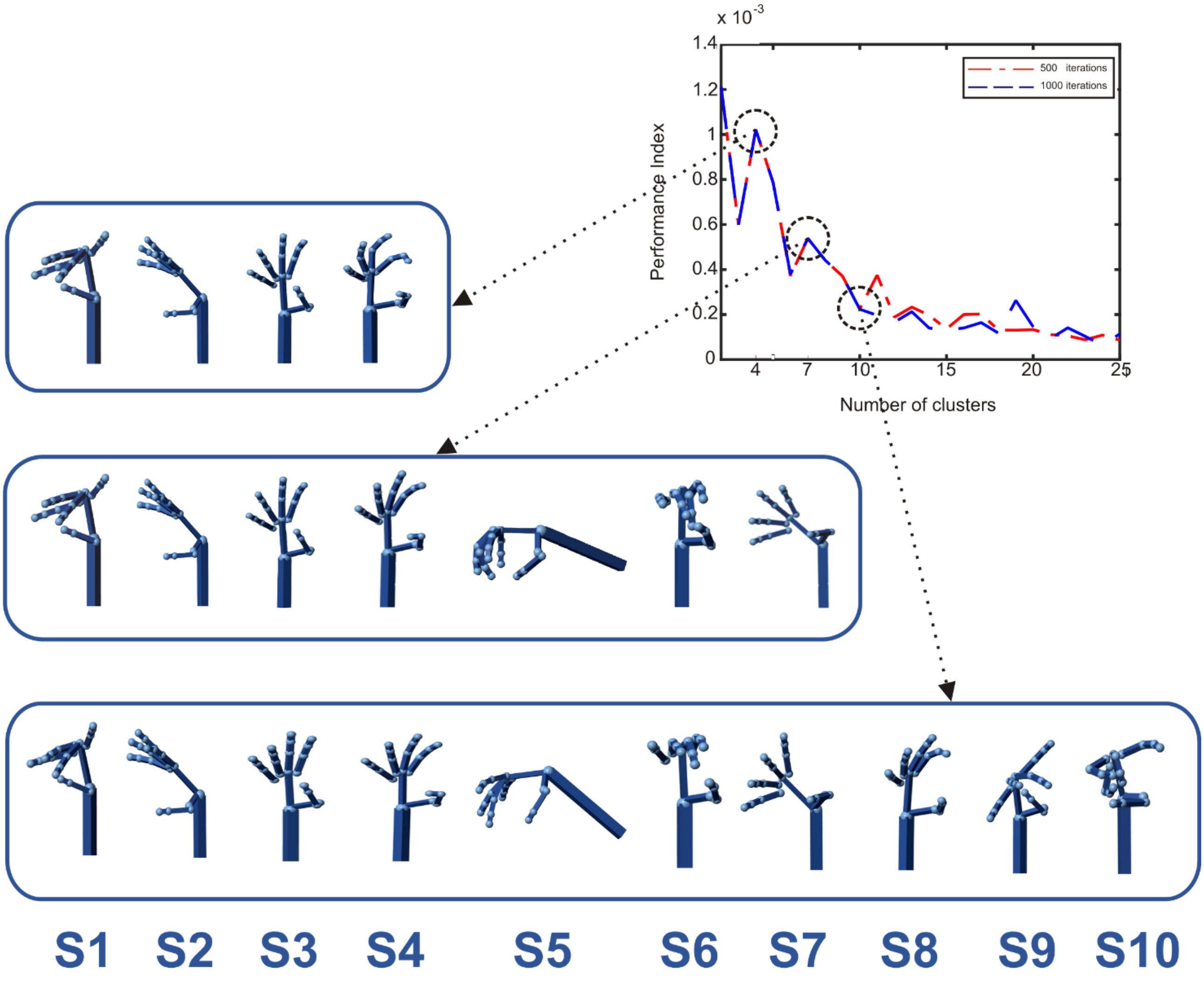

Figure S4. Performance index analysis to assess the optimal number of classes. The performance index as a function of the number of clusters is reported. On the left, we report different hand states obtained at 4 (first row), 7 (second row) and 10 (third row) classes. These 3 sets of hand shapes correspond to the first three peaks of the performance index. These were obtained with 500 (red line) and 1000 (blue line) K-means iterations. Although the absolute maximum of the index suggests 4 clusters, we observed that the solution corresponding to 7 clusters provided us with three additional functional movements. If we consider the next index peak at 10

clusters, this solution gives additional states which are either a repetition of existing ones (S8 - previous S4 in the 7-classes solution) or noise (non-functional hand shapes such as S9, S10). For this reason, in what follows, we selected the solution corresponding to 7 clusters.

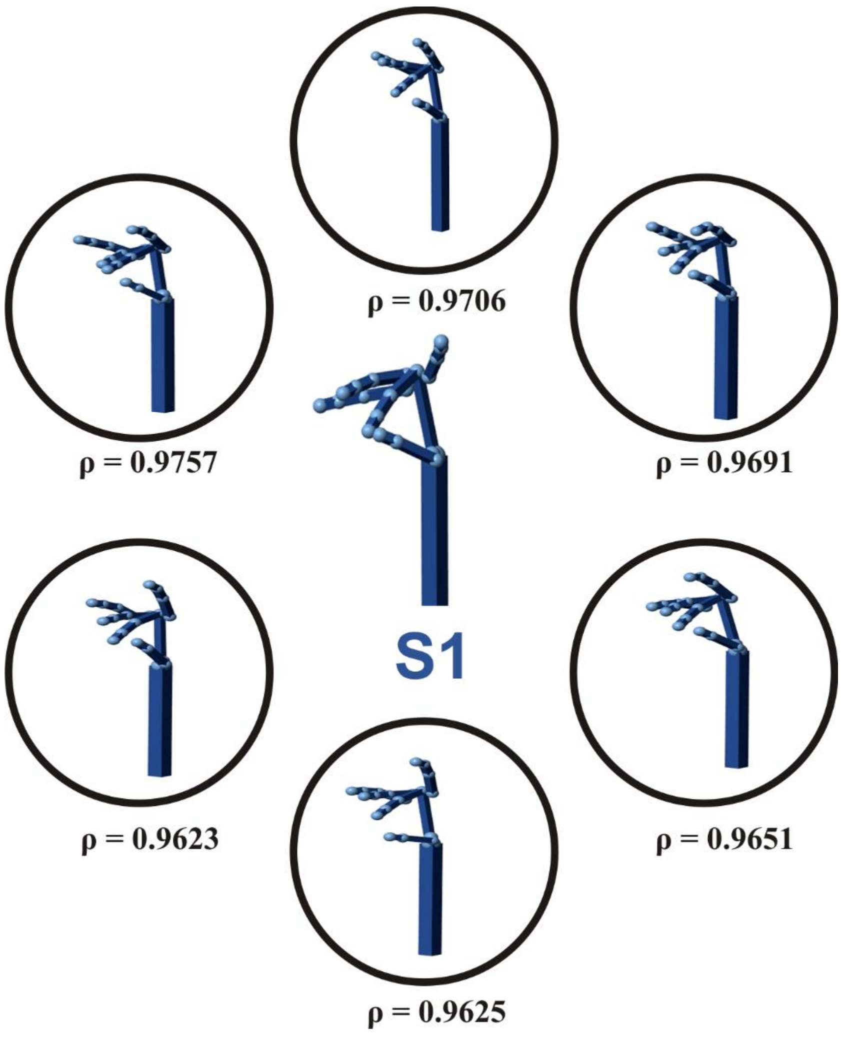

Figure S5. Variability of hand configurations within cluster S1. To interpret more accurately S1**,** we show the variability of the hand configurations within its cluster. Specifically, we show the first six configurations ranked by their correlation (ρ) with the centroid. It can be noted a certain excursion from the average for the index and little finger positions, while the motor configuration of the middle finger is consistent.

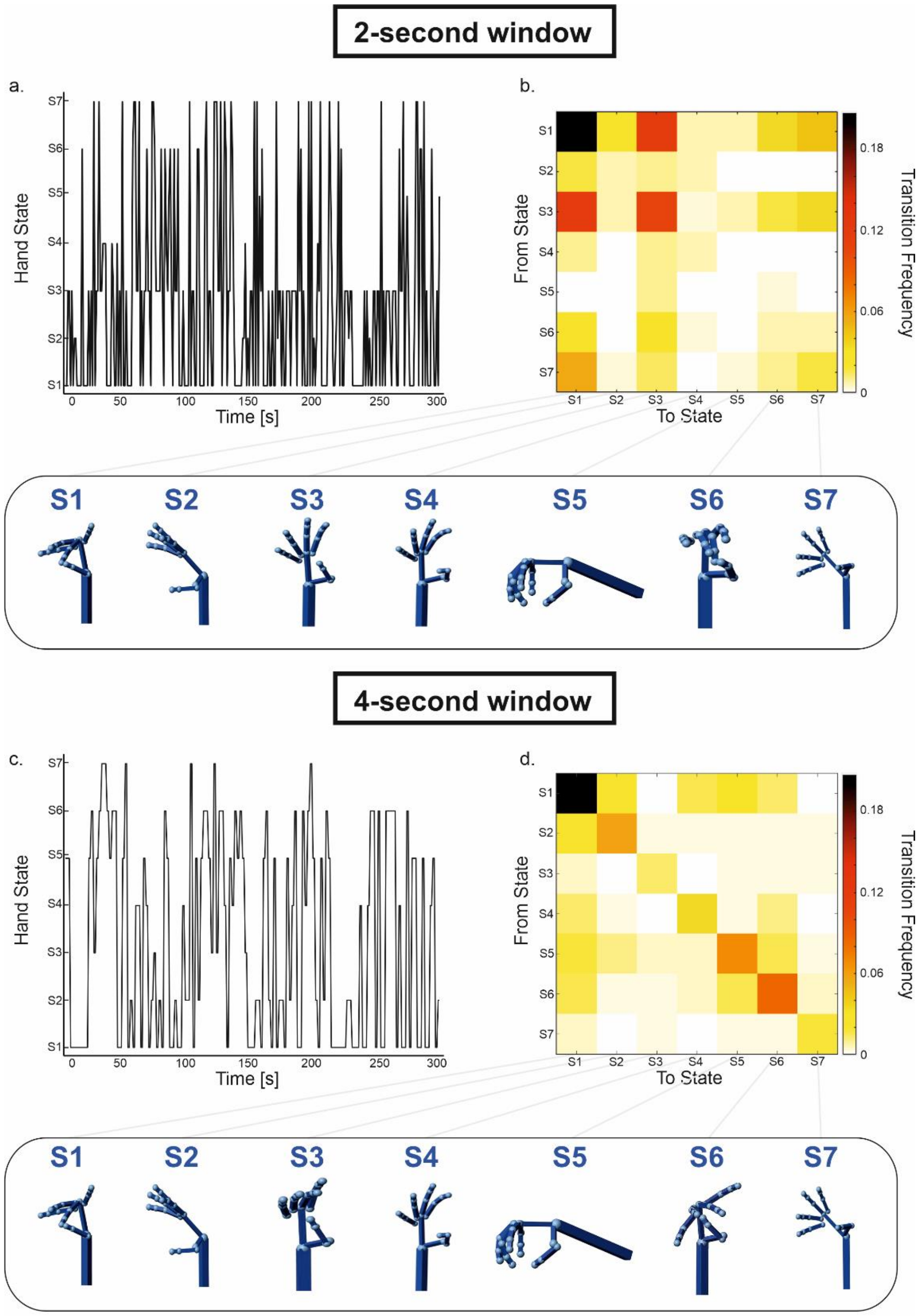

Figure S6. Hand state transitions – windowing test. The previous pipeline was replicated with a larger windowing (4s) to verify the consistency of the previous findings. Spatially, with a 4s window, most of the previous patterns are well reproduced. Temporally, there is a gradual reduction in the temporal resolution of the dynamics, resulting in the loss of important transitions between

states and an enhancement of the internal state dwelling time (see diagonal elements in the transition matrix).

OBJECT	DIAMETER OR WIDTH (cm)	HEIGHT (cm)	WEIGHT (g)
toothpick	0.2	6.5	0.1
coffee cup (handle)	0.9	3.5	73
teaspoon	1	12	2
knife	1	16	3
spoon	1.2	17	3
fork	1.2	18	5
cup (handle)	1.7	6.5	426
egg	2.2	7	44
jug (handle)	2.9	13	95
frying pan (handle)	3	16	270
salt container	4	9	100
sachet of sugar	4.4	7	6
tea bag	4.5	5.5	2
glass	7	8.5	2
bottle of water	7	35	20
packet of cigarettes	7	5	4
circular ashtray	7.4	4.3	72
jar	8	7.5	193
napkin	19.5	19.5	4
plate	22	1.5	12
placemat	43	30	126

Table S1. Everyday objects used by participants. The diameter (or width for non-cylindrical objects), height and weight are reported. Items are sorted according to increasing diameter.

	Max	Min	Range	Mean	Median	SD	Skew
T-MJ	91	-22	113	45	46	17	-0.4
T-MCPJ	23	-31	55	-0.1	0.2	8	-0.3
T-DIPJ	52	-55	107	-5	-6	14	0.2
T-ABD	70	-17	87	43	45	13	-0.8
I-MCPJ	63	-24	87	28	30	16	-0.5
I-PIPJ	105	-6	111	43	41	21	0.4
M-MCPJ	108	-35	143	52	56	26	-0.5
M-PIPJ	84	-4	88	45	47	17	-0.3
M-I ABD	55	-5	60	13	11	8	1.4
R-MCPJ	105	-34	140	50	51	26	-0.3
R-PIPJ	83	-0.7	82	48	50	16	-0.4
R-M ABDJ	57	-0.4	58	19	17	8	1.2
L-MCPJ	91	-24	114	38	37	22	0.1
L-PIPJ	98	-1	99	54	55	18	-0.2
L-R ABDJ	43	5	37	15	15	5	1.6
WP	33	-69	102	-9	-8	14	-0.5
WY	43	-28	71	-1.4	-3	10	0.8

Table S2. Descriptive statistics. Basic kinematic statistics of the sensors associated with the 17 degrees of freedom of the hand. These are the metacarpal-phalangeal (MCP), proximal interphalangeal (PIP) and distal interphalangeal (DIP) joint angles for the four fingers (Index: I, Middle: M, Ring: R, Little: L); the three abduction angles between the four fingers (M-I ABD, R-M ABD, L-R ABD); metacarpal-phalangeal rotation (T-M), metacarpal-phalangeal (T-MCP) and distal interphalangeal (T-DIP) joint angles for the thumb (T) and the abduction angle (T-ABD) between the thumb and the palm of the hand. Finally, the yaw (WY) and pitch (WP) angles of the wrist. Values are averaged across subjects.

PICTURES	ANGLES MEASURED
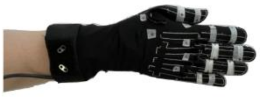	For all abduction (ABD), metacarpophalangeal joint (MPJ), proximal nterphalangeal joint (PIJ) , wrist, and palmar arch sensors, the angular value is set to 0°.
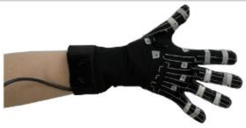	The abduction (ABD) and thumb interphalangeal joint (TIPJ) angles are measured.
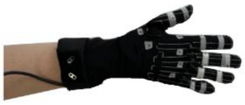	The abduction (ABD) and the thumb metacarpophalangeal joint (TMPJ) angles are measured.
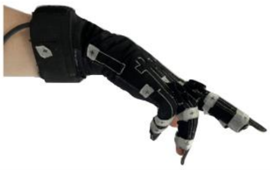	The metacarpophalangeal and the proximal interphalangeal joint angles of the ring (RMCP,RPIP) and little finger (LMCP, LPIP) and the flexion of the wrist (WP) angle are measured.
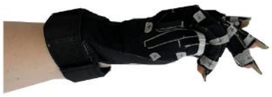	The angles of the metacarpophalangeal and the proximal interphalangeal joint of the index (IMCPJ, IPIPJ) and middle fingers (MMCPJ, MPIPJ) are measured.
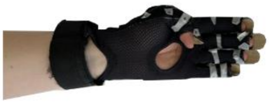	Metacarpophalangeal and interphalangeal joint angles of the thumb (TMCPJ, TIPJ) are measured.
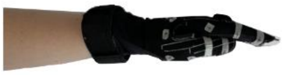	The angle of rotation of the thumb around the palm (TMJ) is measured.
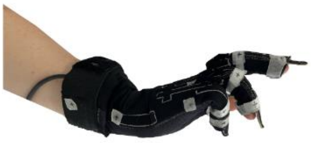	The metacarpophalangeal and the proximal interphalangeal joint angles of the ring (RMCP, RPIP) and little finger (LMCP, LPIP) and the extension of the wrist (WP) angle are measured.
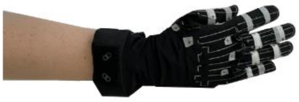	The angle of radial deviation of the wrist is measured (WY).
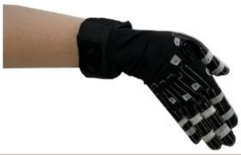	The angle of ulnar deviation of the wrist is measured (WY).

Table S3. Angles measured for each reference hand posture.

Bibliography

1. Ingram, J. N., Körding, K. P., Howard, I. S. & Wolpert, D. M. The statistics of natural hand movements. *Exp Brain Res* **188**, 223–236 (2008).

2. Castiello, U. The neuroscience of grasping. *Nat Rev Neurosci* **6**, 726–736 (2005).
